# Supplementary material for: Accelerated Aging Induced by an Unhealthy High-Fat Diet: Initial Evidence for the Role of Nrf2 Deficiency and Impaired Stress Resilience in Cellular Senescence
Source: Nutrients. 2024 Mar 26;16(7):952. doi: 10.3390/nu16070952 (PMC11013792; doi:10.3390/nu16070952)
Supplement: Supplementary file 1 [file nutrients-16-00952-s001.zip › nutrients-2761211-supplementary.pdf]

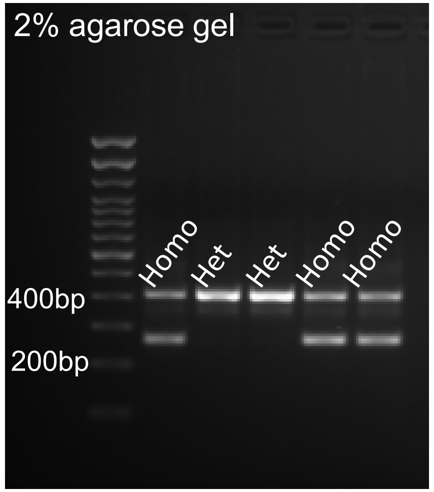

Nrf2 Homozygote = ~400 bp
Nrf2 Heterozygote = ~400 bp and 262 bp

Figure S1: Representative pictures of agarose gel electrophoresis for genotyping Nrf2 transgenic mice. The Nrf2 homozygous knock out were identified with a mutant band at 400bp while the Nrf2 heterozygous mice expressed both the WT and the mutant bands at 262 and 400 bp, respectively.

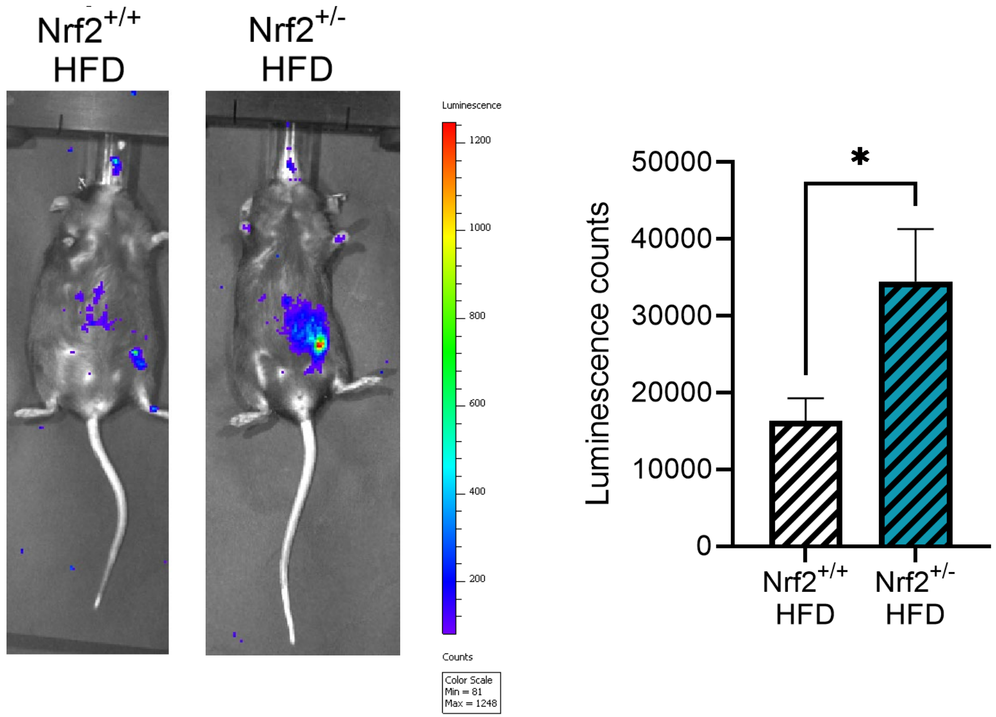

Figure S2. Partial Nrf2 loss increases whole-body senescence. A) Mice were injected with coelentarazine, and luminescence was quantified using a Xenogen IVIS Imaging system 25 minutes after the injection with a 5 minute exposure time. Representative images from $Nrf2^{+/+}$ and $Nrf2^{+/-}$ mice on HFD are shown. B) Quantification of luminescence counts indicates a significant increase in senescent cell burden in $Nrf2^{+/-}$ mice compared to $Nrf2^{+/+}$ mice on HFD (n=5/group). Data are presented as mean±SEM. * indicates a significant difference ($p<0.05$) from the indicated group.
